# NETosis Secondary to the Use of Levamisole-Adulterated Cocaine: A Likely Underlying Mechanism of Vasculopathy

**DOI:** 10.1155/2024/7388799

**Published:** 2024-02-22

**Authors:** Manuela Osorio, Isabel Velásquez, Ruben Vargas, Adriana Vanegas-García, Mauricio Rojas, Gloria Vásquez, Carlos Muñoz-Vahos

**Affiliations:** ^1^Grupo de Inmunología Celular e Inmunogenética, Facultad de Medicina, Universidad de Antioquia, Medellín, Colombia; ^2^Hospital Universitario San Vicente Fundación, Medellín, Colombia; ^3^Sección de Reumatología, Facultad de Medicina, Universidad de Antioquia, Medellín, Colombia

## Abstract

**Background:**

Since 2010, several cases of a new vasculopathy induced by the use of levamisole-adulterated cocaine (LAC) have been reported. This vasculopathy is characterized by retiform purpura, earlobe necrosis, multisystem compromise, and multiple autoantibodies. Given its similarity to antineutrophil cytoplasmic antibody (ANCA)-associated vasculitis, LAC-associated vasculopathy is postulated to be mediated by pathophysiologic processes resulting from neutrophil cell death by NETosis, a phenomenon previously described in ANCA vasculitis. This study tries to establish the presence of NETosis induced by cocaine, levamisole, or both. *Methodology*. Neutrophils were isolated from the peripheral blood of healthy controls by Ficoll-Hystopaque density gradient centrifugation followed by dextran sedimentation. Cell viability and purity were evaluated by flow cytometry after staining with PI/DiOC6 and labeling with fluorescent anti-CD45/anti-CD3 monoclonal antibodies (mAbs), respectively. Neutrophils were exposed to levamisole, cocaine, a cocaine-levamisole mixture, and sera pools from healthy controls and patients with LAC-associated vasculopathy. NETosis was then assessed by flow cytometry after staining cells with Sytox Green, Hoechst-33342, and fluorescent antineutrophil elastase (NE) and antimyeloperoxidase (MPO) mAbs. In addition, NETosis was morphologically confirmed by fluorescence microscopy. Proinflammatory cytokine levels in culture supernatants and reactive oxygen species (ROS) synthesis were determined by flow cytometry. The involvement of calcium and muscarinic receptors in cell death induction was evaluated in parallel experiments carried out in the presence of 1,2-bis (o-aminophenoxy) ethane-N, N, N′, N′-tetraacetic acid (BAPTA) and hyoscine butylbromide (HBB), their respective inhibitors.

**Results:**

Cocaine, levamisole, and a cocaine-levamisole mixture induced neutrophil cell death. DNA/MPO extrusion and cell morphology patterns were consistent with NETosis. Neither proinflammatory cytokines nor ROS behaved as proNETotic factors. Preliminary results suggested that muscarinic receptors and calcium-dependent signals were involved in LAC-induced NETosis.

**Conclusions:**

Cocaine, levamisole, and a cocaine-levamisole mixture can induce NETosis through mechanisms involving muscarinic receptors and calcium-dependent pathways.

## 1. Introduction

Vasculopathies are diseases of the blood vessels that can cause ischemic lesions in different organs, ranging in severity from mild to severe enough to cause death [1]. Inflammation of blood vessels is a type of vasculopathy and comprises several diseases known as vasculitis. Vasculitis are classified according to the caliber of the affected vessels into large artery, medium vessel, and small vessel vasculitis or according to their etiology into primary and secondary vasculitis. Primary vasculitis are those in which no specific triggering stimulus can be identified, while secondary vasculitis are induced by drugs, infections, neoplasms, or autoimmune diseases [2, 3].

Levamisole is an anthelmintic drug with nonspecific immunomodulatory properties. However, in 1999, it was withdrawn from the US market and restricted to veterinary use because its administration was associated with purpuric lesions on both cheeks and necrosis of earlobes [4, 5]. In 2002, the use of levamisole as a cocaine additive was first reported in the US. Such adulteration increased from 1% to 70% in less than a decade [4, 6, 7]. In 2010, several cases of levamisole-adulterated cocaine (LAC)-induced vasculopathy were described. Patients exhibited a variety of clinical manifestations, including painful retiform purpura with or without central necrosis and hemorrhagic blisters, as well as elevated titers of antinuclear autoantibodies (ANAs) and antineutrophil cytoplasmic autoantibodies (ANCAs), including those directed against granule myeloperoxidase (MPO) and proteinase 3 (PR3) [8, 9].

An important group of primary vasculitis comprises ANCA-associated vasculitis (AAVs) [10, 11] characterized by circulating ANCAs directed against MPO and PR3 proteins of neutrophil granules. Neutrophil cell death is implicated in the pathophysiology of AAVs. However, it is essential to note that neutrophils can undergo necrosis, apoptosis, and NETosis. NETosis is a type of death characterized by neutrophil extracellular trap (NET) formation resulting from the extrusion of chromatin and granule proteins. This type of death appears to play a crucial role in the pathogenesis of vasculitis.

Levamisole is known to induce the release of NETs [12]. However, the effect of the cocaine-levamisole combination on NETosis and its implications for the pathophysiology and clinical presentation of LAC-associated vasculitis is unknown.

NETosis can be induced by different mechanisms such as the activation of muscarinic receptors, the production of ROS—catalyzed by the NADPH-oxidase complex—or the increase in intracellular calcium levels, which in turn activates the enzyme peptidyl-arginine deiminase 4 (PAD4) [13]. Although some studies have described the underlying mechanism of levamisole-induced NETosis, the mechanism by which the cocaine-levamisole combination could induce NETosis is still unknown.

Understanding the underlying mechanisms of LAC-associated vasculitis could provide therapeutic options, as current treatment is based on immunosuppressants, which are related to various adverse events. For example, as levamisole activates muscarinic receptors expressed on neutrophils, treatments could be designed to block muscarinic receptor-dependent signaling by inhibitors such as hyoscine butylbromide (HBB).

## 2. Materials and Methods

### 2.1. Reagents

RPMI-1640 culture medium, fetal bovine serum bovine (FBS), Dulbecco's phosphate buffered saline (PBS), and Dextran 500 were purchased from Gibco™, Thermo Fisher Scientific Inc. (Waltham, MA, USA). Histopaque-1077, trypan blue, levamisole hydrochloride (C11H12N2S-HCl) and hyoscine butylbromide (catalogue number, H1450000), phorbol 12-myristate 13-acetate (PMA), and dihydrorhodamine 123 (DRH123) were obtained from Millipore Sigma (Burlington, MA, USA). The Cytometric Bead Array (CBA) Human Inflammatory Cytokine kit, and the anti-CD45-PeCy7 mAb (clone 30-F11) were purchased from Becton Dickinson (BD) Biosciences (San Diego, CA, USA). 3,3′-Dihexyloxacarbocyanine iodide (DIOC_6_) and propidium iodide (PI) were bought from Invitrogen™ Thermo Fisher Scientific Inc. (Waltham, MA, USA). Sytox Green and Hoechst 33342 were acquired from Thermo Fisher Scientific. The anti-MPO-PE (clone 2C7) and antineutrophil elastase (NE)-Alexa fluor 647 (clone 950317) mAbs were purchased from Novus Biologicals Biotechne (Littleton, CO, USA). Cocaine.HCI (1.0 mg base/1 mL methanol) (analytical reference standard COC-156-HC-1LM) was purchased from Lipomed AG (Arlesheim, Basel-Country, Switzerland) and imported via Químicos® complying with all permissions of the Fondo Nacional Antidrogas (FONA) of Colombia.

### 2.2. Patients and Controls

This study included patients with a confirmed diagnosis of LAC-associated vasculitis (*n* = 25) who were evaluated by the Rheumatology Group of the University of Antioquia (GRUA, for its acronym in Spanish) at the Rheumatology Service of the Hospital Universitario San Vicente Fundación in Medellín. Patients were classified into two groups, those with glomerulonephritis (GN) and those without GN, according to their clinical information. Healthy individuals matched by sex and similar in age to the group of patients were also included as controls (*n* = 10). All individuals were of legal age, agreed to participate in the study voluntarily, and signed the informed consent form. Exclusion criteria were active infection at blood sampling, diagnosis of malignant neoplasia, and the impossibility of accessing the clinical or paraclinical records necessary for the analyses proposed in the study.

### 2.3. Preparation of Pooled Sera

Peripheral blood samples (6 mL) were drawn from patients and healthy controls. Blood samples were allowed to clot at room temperature (RT) and then centrifuged at 900 × g 10 min at RT to separate the serum. Serum samples were aliquoted and stored at −20°C until use. Pooled sera were prepared from samples from healthy controls, patients with GN, and patients without GN.

### 2.4. Isolation of Neutrophils

**Figure 1:**
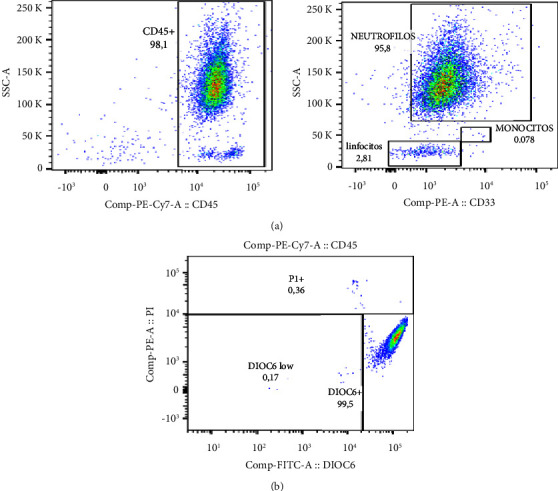
Purity and viability of neutrophil-enriched suspensions. Circulating neutrophils from healthy controls were isolated by Ficoll-Hystopaque density gradient centrifugation followed by dextran sedimentation. Cell purity and viability were evaluated by labeling with anti-CD45-PE/Cy7 and anti-CD33-PE mAbs and PI/DiOC_6_ staining, respectively. (a) Representative dot plots showing the strategy for gating CD44^+^CD33^+^ neutrophils and evaluating cell purity. (b) Representative dot plot illustrating the distinction of live cells (DiOC_6_^bright^ IP^neg^), cells with mitochondrial injury (DIOC_6_^dim^IP^neg^), and cells with compromised plasma membrane (DIOC_6_^neg^IP^+++^). *n* = 4 independent experiments.

Peripheral blood samples (8 mL) were drawn from healthy controls in tubes containing acid citrate dextrose (ACD) anticoagulant. The blood sample was brought to a final volume of 10 mL with 1X PBS. This mixture was gently added to the walls of a 15 mL tube containing 3 mL of Hystopaque-1077 and centrifuged at 900 × g for 30 min at RT. Then, the top three layers (plasma, mononuclear cell, and isolation media) were removed. The bottom layer containing erythrocytes and neutrophils was suspended in PBS to a volume of 7 mL, mixed with an equal volume of 3% dextran, and incubated at RT for 20 min. The neutrophil population was then carefully removed and transferred to a 15 mL tube, resuspended in PBS to complete 14 mL, and centrifuged at 650 × g for 7 min. Then, the supernatant was discarded, and cells were suspended in 5 mL of hypotonic PBS, allowed to stand for 30 s, mixed with 5 mL of re-equilibration PBS, and centrifuged at 650 × g for 7 min. This step was repeated once more. Finally, cells were resuspended in 600 *µ*L of RPMI without SBF, and their count was determined in a Neubauer chamber using the trypan blue dye exclusion method. Then, 100,000 cells were suspended in 200 µL of RPMI to determine their viability and purity in BD LSRFortessa™ (BD Biosciences). Viability was assessed by staining with DIOC_6_ and PI and purity by labeling with anti-CD45-PE/Cy7 and anti-CD33-PE mAbs for 10 minutes at room temperature. Viability was ≥95%, and purity reached values above 93% (Figures 1(a) and 1(b)).

### 2.5. Induction and Assessment of NETosis

**Figure 2:**
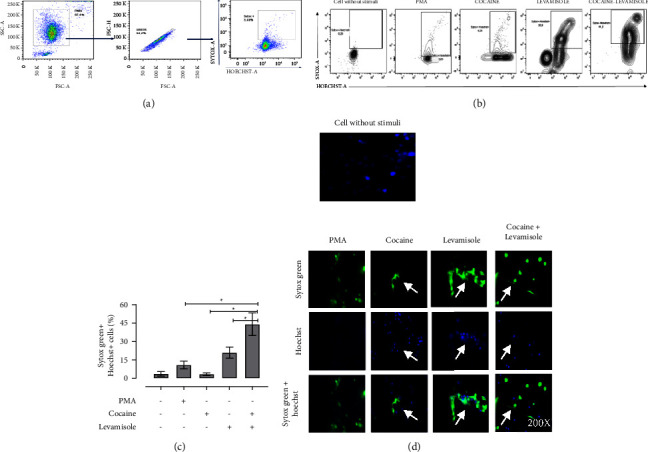
Induction of NETosis in neutrophil cultures exposed to cocaine, levamisole, and cocaine-levamisole mixture. Neutrophil-enriched suspensions from healthy controls were exposed to cocaine (40 *µ*M), levamisole (20 nM), cocaine-levamisole mixture (20 *µ*M/40 nM), and PMA (20 nM) for 6 h at 37°C. Staining with Sytox Green, Hoechst 33342, and mAbs anti-MPO-PE and anti-NE-Alexa fluor 647 was used to evaluate extrusion of neutrophil DNA and granular content. (a) Representative dot plots illustrating the strategy for gating double positive Sytox Green^+^ Hoechst 33342^+^ netting neutrophils. (b-c) Representative dot plots and column chart showing the percentage of netting neutrophils in response to each treatment. Bar represents median values and error bars correspond to IQRs. ^*∗*^*p* ≤ 0.05; Kruskal–Wallis test. *n* = 4 independent experiments. (d) Immunofluorescence analysis by confocal microscopy confirming NET formation. Magnification: 200x. *n* = 3 independent experiments.

Preliminary assays were carried out to establish experimental conditions to induce and evaluate NETosis. To this purpose, neutrophil-enriched suspensions were exposed to cocaine (20 *µ*M and 40 *µ*M), levamisole (20 nM and 40 nM), a cocaine-levamisole mixture (20 *µ*M/40 nM), and the pooled sera from patients with LAC-associated vasculitis (5%, 10%, 20%, and 40%) for 2, 4, and 6 h (data not shown). From these preliminary results, the following protocol was set: in brief, 1 × 10^5^ neutrophils/200 *µ*L of RPMI were placed in 12 × 75 mm tubes and stimulated for 6 h, at 37°C, with cocaine (20 *µ*M), levamisole (40 nM), a cocaine-levamisole mixture (20 *µ*M/40 nM), and pooled sera (20%) from healthy controls (PHCs), and patients with LAC-associated vasculitis and GN (PWN) or without GN (PWON). A parallel assay was carried out with PMA (20 nM) as cell death control. Supernatants were collected and kept at −80°C until analysis. Cells were stained with Sytox Green, Hoechst 33342, and anti-MPO-PE and anti-NE-Alexa fluor 647 mAbs for 10 minutes at 37°C and acquired on a BD LSRFortessa™ (BD Biosciences). Data were analyzed using the FlowJo software. According to Zharkova et al. [14], netting neutrophils were evidenced as double-positive Hoechst 33342^+^ and Sytox Green^+^ events. These two dyes stain nucleic acids but differ because Hoechst 33342 crosses the cell membrane and Sytox Green does not, so it will only bind to nucleic acids when the cell membrane is damaged and the genetic material is exposed (Figure 2(a)).

### 2.6. Inhibition of NETosis

Neutrophil-enriched suspensions (1 × 10^5^ cells/200 *µ*L of RPMI) were placed in 12 × 75 mm tubes and incubated with 20 *µ*L of 20 nM HBB (concentration established from pilot assays) for 10 min. Cells were then exposed to cocaine (20 *µ*M), levamisole (40 nM), a levamisole-cocaine mixture (20 *µ*M/40 nM), PHC, PWN, and PWON pooled sera (20%) and PMA (20 nM), as positive cell death control, for 6 h at 37°C. Supernatants were collected and kept at −80°C until analysis. After, cells were stained as described above to evaluate NETosis by flow cytometry.

### 2.7. Quantification of Proinflammatory Cytokines

The levels of the proinflammatory cytokines IL-12p70, TNF, IL-10, IL-6, IL-1*β*, and IL-8 were quantified in culture supernatants by flow cytometry using the Cytometric Bead Array (CBA) kit, following the manufacturer's instructions. In brief, the supernatants were incubated with six populations of microspheres with different fluorescence intensities coated with capture mAbs targeting the different cytokines. They were then incubated with PE-labeled detection mAbs for 3 h, washed with wash buffer, and centrifuged at 200 × g for 5 min. Finally, supernatants were discarded, and the beads were resuspended in the wash buffer, acquired on an BD LSRFortessa™ (BD Biosciences), and analyzed with the FlowJo software.

### 2.8. Analysis of NETosis by Fluorescence Microscopy

Neutrophil-enriched suspensions (1 × 10^5^ cells/200 *µ*L of RPMI) were allowed to adhere to the bottom of Falcon™ 24-well culture plates (catalogue number 353047) at 37°C for 30 min. Cells were then exposed to cocaine (20 *µ*M), levamisole (40 nM), a cocaine-levamisole mixture (20 *µ*M/40nM), PHC, PWN, and PWON pooled sera (20%) and PMA (20 nM), as a positive cell death control, for 6 h at 37°C. After incubation, cells were stained with Sytox Green, Hoechst 33342, and anti-MPO-PE mAb. The extrusion of NETs was evaluated on a fluorescence microscope. Image analysis was performed with ImageJ software.

### 2.9. Generation of Reactive Oxygen Species

Neutrophil-enriched suspensions (1 × 10^5^ cells/500 *µ*L RPMI in 12 × 75 mm tubes) were prewarmed at 37°C for 5 min and then incubated with dihydrorhodamine 123 (1 : 1000) at 37°C for 10 min. Cells were then exposed to cocaine (20 *µ*M), levamisole (40 nM), a cocaine-levamisole mixture (20 *µ*M/40 nM), and PMA (20 nM), as a positive control of ROS generation, for 20 min and 60 min. After incubation, cells were placed on ice for 10 min to stop the reaction and washed twice with 1X PBS and centrifugation at 450 × g for 5 min at 4°C. Subsequently, supernatant was removed, and cells were resuspended in PBS and kept on ice until acquisition on the BD LSRFortessa™ (BD Biosciences). Data were analyzed using the FlowJo software.

### 2.10. Inhibition of Calcium-Dependent Pathways

Neutrophil-enriched suspensions (1 × 10^5^/cell vol of 200 *µ*l 12 × 75 mm tubes) were incubated with BAPTA, a calcium chelator (0, 2 *µ*M, and 20 *µ*M) for 10 min and then exposed to a cocaine-levamisole mixture (20 *µ*M/40 nM) for 3 h. After incubation, cells were stained with Sytox Green and Hoechst 33342, washed, and acquired on the BD LSRFortessa™ (BD Biosciences). Data were analyzed with the FlowJo software.

### 2.11. Statistics

Demographic and clinical characteristics of patients and healthy controls are described as medians and percentages. Results from different treatments were compared with the Kruskall–Wallis test followed by Dunn's test for multiple comparisons. Biological replicates (*n* = 2–8) of each assay were independently carried out and are indicated in the corresponding figures. Data are shown in column charts (medians and interquartile ranges). Differences were considered significant when *p* < 0.05. Statistical analyses were run in the GraphPad Prism 8.0.1 software.

## 3. Results

### 3.1. Demographic and Clinical Characteristics of Patients

The present study included serum samples from 25 out of 30 patients with LAC-associated vasculitis enrolled in the cohort study previously described by Muñoz-Vahos et al. [15]. Their demographic and clinical data are summarized in Table 1. Patients had a median age of 34 years (range 25–51), were primarily male (21/25; 84%), and most reported having consumed substances other than cocaine, such as marijuana (19/25), tobacco (19/25), and alcohol (16/25). The most frequent clinical manifestations were necrosis of earlobes (20/25; 80%), arthralgias/arthritis (16/25; 64%), and histopathologically confirmed nephritis (15/25; 60%), with a predominance of immune complex-mediated proliferative GN. Skin ulcers (9/25; 36%) and autoimmune hemolytic anemia (7/25; 28%) were also observed. Histopathological study of skin biopsies showed pseudovasculitis (4/13; 30%), pyoderma gangrenosum (3/13; 23%), leukocytoclastic vasculitis (2/13; 16%), thrombotic vasculopathy (2/13; 16%), and the two latter types of vascular involvement (2/13; 16%). In addition, neutropenia (absolute neutrophil count <1500/*μ*L) (4/25; 16%), C3 hypocomplementemia (13/25; 52%), C4 hypocomplementemia (3/25; 12%), positive ANA test (14/25; 56%), positive ANCA test (21/25; 85%) with perinuclear pattern (9/21, 43%), positive anti-MPO antibodies (23/25; 92%), and positive anti-PR3 (7/25; 28%) antibodies were observed.

### 3.2. Induction of NETosis in Neutrophil Cultures Exposed to Cocaine, Levamisole, And Cocaine-Levamisole Mixture

**Figure 3:**
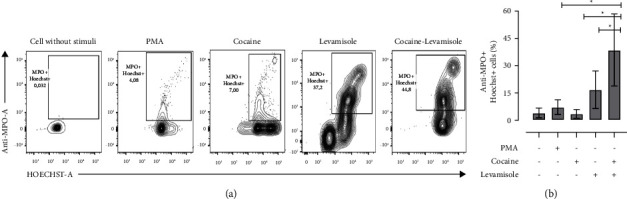
Evaluation of MPO in NETs extruded from cultures of neutrophils exposed to cocaine, levamisole, and cocaine-levamisole mixture. Neutrophil-enriched suspensions from healthy controls were exposed to cocaine (20 *µ*M), levamisole (40 nM), cocaine-levamisole mixture (20 *µ*M/40 nM), and PMA (20 nM) for 6 h. Staining with Sytox Green, Hoechst 33342, and anti-MPO-PE and anti-NE-Alexa fluor 647 mAbs was used to evaluate extrusion of neutrophil DNA and granular content. (a) Representative dot plots and (b) column chart showing the percentages of double positive Hoechst 33342^+^ MPO^+^ netting neutrophils generated in response to each treatment. Bar represents median values and error bars correspond to the IQRs. ^*∗*^*p* ≤ 0.05; Kruskal–Wallis test. *n* = 4 independent experiments.

When neutrophil-enriched suspensions from healthy controls were exposed to cocaine, levamisole, and a cocaine-levamisole mixture, a higher proportion of double-positive Sytox Green^+^Hoeschst-33342^+^ cells were observed (median = 20.28% and IQR: 8.02–44.7) than in suspensions exposed to cocaine (median = 2.52% and IQR: 0.443–3.35). Notably, the cocaine-levamisole mixture showed a synergistic effect on NETosis induction (median = 67.43% and IQR: 31.3–88.8) (Figures 2(b) and 2(c)). The NET formation was confirmed by confocal microscopy. Indeed, Hoechst-33342 and Sytox Green signals colocalized on NETs, supporting the induction of neutrophil cell death via NETosis (Figure 2(d)). In addition, cytometry analyses showed that the percentage of double-positive Hoechst-33342^+^ MPO^+^ cells was higher in cultures treated with levamisole (median = 13.7% and IQR: 8.22–31.70) than in those exposed to cocaine (median = 2.7% and IQR: 1.69–7.00) and much higher in cultures exposed to the cocaine-levamisole mixture (median = 32.6% and IQR: 23.30–65.90). On the other hand, NE was not detected under any of the experimental conditions described. The presence of MPO in netting neutrophils confirmed that cocaine, levamisole, and the cocaine-levamisole mixture induced neutrophil cell death by NETosis (Figure 3(a) and 3(b)).

### 3.3. Induction of NETosis in Neutrophil Cultures Exposed to Pooled Sera from Patients with LAC-Associated Vasculitis

**Figure 4:**
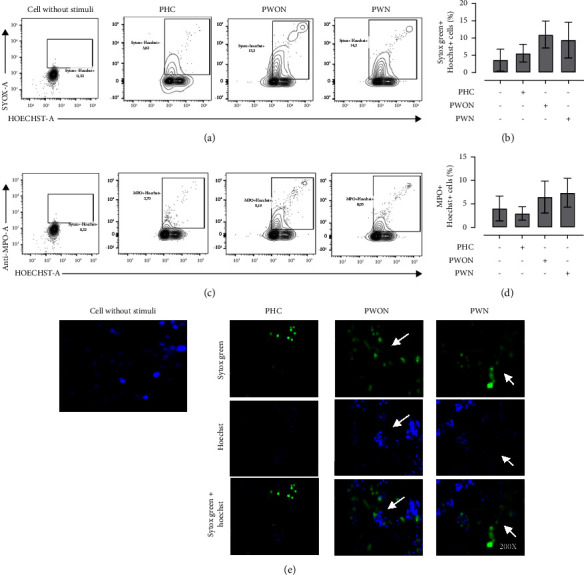
Induction of NETosis in neutrophil cultures exposed to sera from patients with LAC-associated vasculitis. Neutrophil-enriched suspensions from healthy controls were exposed to pooled sera (20%) from healthy controls (PHCs) and patients with LAC-associated vasculitis with GN (PWN) or without GN (PWON) for 6 h at 37°C. Staining with Sytox Green, Hoechst 33342, and anti-MPO-PE and anti-NE-Alexa fluor 647 mAbs was used to evaluate extrusion of neutrophil DNA and granular MPO. (a-b) Representative dot plots and column chart showing the percentages of double positive Sytox Green^+^ Hoechst 33342^+^ netting neutrophils generated in response to each treatment. (c-d) Representative dot plots and column chart showing the percentages of double positive MPO ^+^ Hoechst 33342^+^ netting neutrophils generated in response to each treatment. Bar represents median values and error bars correspond to IQRs. ^*∗*^*p* ≤ 0.05; Kruskal–Wallis test. *n* = 4 independent experiments. (e) Immunofluorescence analysis by confocal microscopy confirming NET formation. Magnification: 200x. *n* = 4 independent experiments.

To evaluate whether soluble serum component from patients with LAC-associated vasculitis could induced NETosis, neutrophil-enriched cell suspensions from healthy controls were incubated with PHC, PWN, and PWON pooled sera. Similar percentages of double positive Sytox Green^+^Hoechst-33342^+^ cells were generated in the presence of PWN (median = 10.6% and IQR: 2.39–14.3) and PWON (median = 11.4% and IQR: 5.97–15.00). These values were lower than those generated by the cocaine-levamisole mixture but higher than those from cultures incubated with PHC (median = 5.1% and IQR: 3.35–8.75) (Figure 4).

### 3.4. Proinflammatory Cytokines in Neutrophil Cultures Exposed to Cocaine, Levamisole, and Cocaine-Levamisole Mixture

To assess whether proinflammatory cytokine accumulation was related to the NETosis induced by the cocaine-levamisole mixture, neutrophil-enriched suspensions from healthy controls were exposed to cocaine, levamisole, and the cocaine-levamisole mixture. Supernatants were collected and kept at −20°C until quantitation of TNF-*α*, IL-1*β*, IL-12p70, IL-8, IL-6, and IL-10 by CBA and flow cytometry. The level of IL-8 showed a tendency to increase in neutrophil cultures exposed to cocaine.

**Figure 5:**
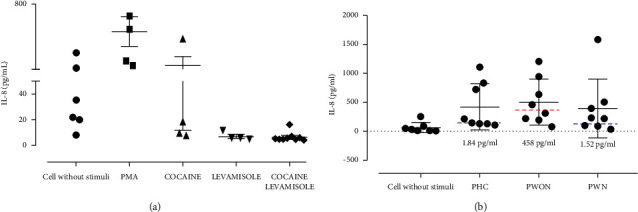
IL-8 levels produced by neutrophil cultures exposed to cocaine, levamisole, and cocaine-levamisole mixture. Supernatants collected from cultures described in Figures 3 and 4 were kept a −80°C. Then, they were thawed to quantify the levels of proinflammatory cytokines. Only IL-8 showed changes. (a) Cocaine, levamisole, and the cocaine-levamisole mixture and (b) pooled sera from healthy controls (PHCs) and patients with LAC-associated vasculitis with (PWN) or without (PWON) GN. The average basal concentration of IL-8 in PHC (violet line), PWON (red line), and PWN (blue line) are shown with their corresponding values. Graphs show median values and IQRs. ^*∗*^*p* ≤ 0.05; Kruskal–Wallis test. *n* = 1 independent experiments.

In contrast, the IL-8 levels increased in neutrophil cultures exposed to different sources of serum PHC (mean basal and stimulated: 1,84–427 pg/mL), PWON (mean basal and stimulated: 458–508 pg/mL), and PWN (mean basal and stimulated: 1,52–399 pg/mL), but no significant difference was observed between the three serum sources (Figure 5).

### 3.5. ROS Production in Neutrophil Cultures Exposed to Cocaine, Levamisole, and Cocaine-Levamisole Mixture

**Figure 6:**
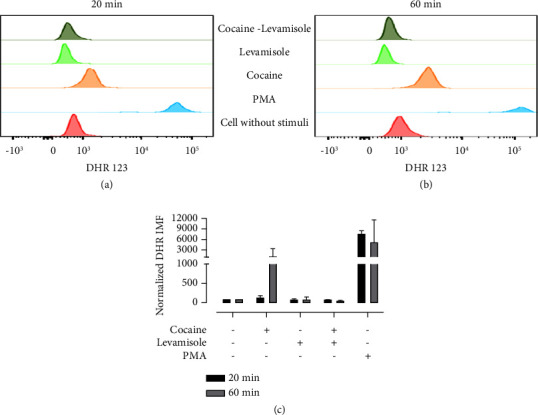
ROS generation in neutrophil cultures exposed to cocaine, levamisole, and cocaine-levamisole mixture. Neutrophil-enriched suspensions were incubated with dihydrorhodamine 123 (1 : 1000) for 10 min before adding cocaine (20 *µ*M), levamisole (40 nM), a cocaine-levamisole mixture (20 *µ*M/40 nM), and PMA (20 nM) for 20 and 60 min, washed, and analyzed by flow cytometer. (a-b) Representative histograms and (c) column chart showing MFI of rhodamine 123 in neutrophils under each treatment. Graphs show median values and error bars correspond to IQRs. ^*∗*^*p* ≤ 0.05; Kruskal–Wallis test. *n* = 3 independent experiments.

To assess whether cocaine and levamisole treatment induced the formation of NETs through the release of ROS, neutrophils were incubated with cocaine, levamisole, and a cocaine-levamisole mixture for 20 and 60 min. Afterwards, DHR123 was added, and its conversion to rhodamine mediated by superoxide anion was assessed by flow cytometry. Cocaine induced a slight increase (1.3x) in ROS production. Interestingly, cultures treated with levamisole showed lower ROS synthesis than the negative control. Moreover, ROS production was lowered in cultures exposed to the cocaine-levamisole mixture than to cocaine (Figure 6), suggesting an antagonistic effect of levamisole on cocaine-induced ROS production and that it might not be involved in the induction of NETosis (Figure 6).

### 3.6. Role of Intracellular Calcium in NETosis Induced by a Cocaine-Levamisole Mixture

**Figure 7:**
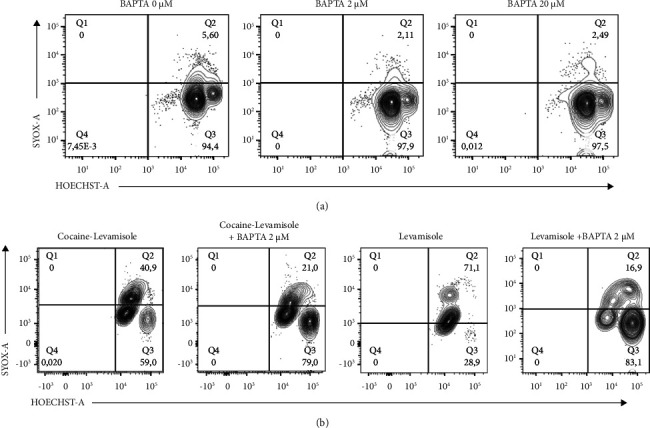
Role of intracellular calcium in NETosis induced by a cocaine-levamisole mixture. Neutrophil-enriched suspensions from healthy controls were incubated with BAPTA at different concentrations for 10 min before adding levamisole (40 nM) and the cocaine-levamisole mixture (20 *µ*M/40 nM) for 3 h. After incubation, cells were stained with Sytox Green and Hoechst 33342, washed, and analyzed by flow cytometry. (a) Representative dot plots showing double positive Sytox Green^+^ Hoechst 33342^+^ netting neutrophils in presence of BAPTA. (b) Representative dot plots showing the effect of 2 *µ*M BAPTA on reduction of double positive Sytox Green^+^ Hoechst 33342^+^ netting neutrophils induced in the presence of the cocaine-levamisole mixture or levamisole. *n* = 2 independent experiments.

The increase in intracellular calcium level is one of the mechanisms involved in the induction of NETosis since it activates the enzyme PAD4. This enzyme participates in chromatin decondensation and subsequent NET extrusion from neutrophils. To determine whether the NETosis induced by the cocaine-levamisole mixture was due to changes in the intracellular calcium level, neutrophils were preincubated with BAPTA (2 *μ*M and 20 *μ*M), a calcium chelator, for 3 h before adding cocaine, levamisole, and a cocaine-levamisole mixture. The percentage of netting neutrophils was 40% in cultures treated with the cocaine-levamisole mixture and decreased to MFI 21% in the presence of BAPTA (Figure 7). These results suggest that cocaine and levamisole combined could induce NETosis by regulating intracellular calcium levels; however, further assays are needed to confirm this hypothesis.

### 3.7. Role of Muscarinic Receptors in NETosis Induced by a Cocaine-Levamisole Mixture

**Figure 8:**
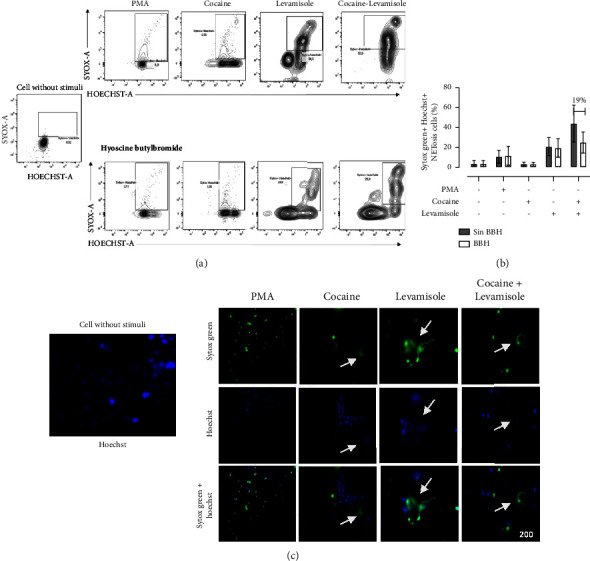
Role of muscarinic receptors in NETosis induced by a cocaine-levamisole mixture. Neutrophil-enriched suspensions were incubated with 20 nM HBB for 10 min and then exposed to cocaine (20 *μ*M), levamisole (40 nM), and the cocaine-levamisole mixture (20 *µ*M/40 nM) and PMA (20 nM) for 6 h at 37°C. After incubation, cells were stained with Sytox Green and Hoechst 33342, washed, and analyzed by flow cytometry. (a-b) Representative dot plots and column chart showing the percentages of double positive Sytox Green^+^ Hoechst 33342^+^ netting neutrophils induced by each treatment. Bars show median values and error bars correspond to IQRs. ^*∗*^*p* ≤ 0.05; Kruskal–Wallis test. *n* = 4 independent experiments. (c) Immunofluorescence analysis by confocal microscopy confirming the inhibition of NET formation by HBB. Magnification: 200x. *n* = 4 independent experiments.

Hyoscine butylbromide is a quaternary ammonium compound with an anticholinergic effect. It has an antagonistic effect on muscarinic receptors present in different cells, including neutrophils [16]. To determine whether NETosis induced by cocaine, levamisole, and the cocaine-levamisole mixture involved muscarinic receptors, neutrophils were incubated with 20 *µ*L of HBB (20 nM) for 10 min before exposure to cocaine, levamisole, and a cocaine-levamisole mixture for 6 h at 37°C. After incubation, cells were stained with Sytox Green and Hoechst 33342, washed, and analyzed by flow cytometry. BBH reduced the frequency of double positive Sytox Green^+^ Hoechst^+^ netting neutrophils by MFI 18.9%. This reduction was confirmed by fluorescence microscopy (Figures 8(a)–8(c)).

### 3.8. Role of Muscarinic Receptors in NETosis Induced by Sera from Patients with LAC-Associated Vasculitis

**Figure 9:**
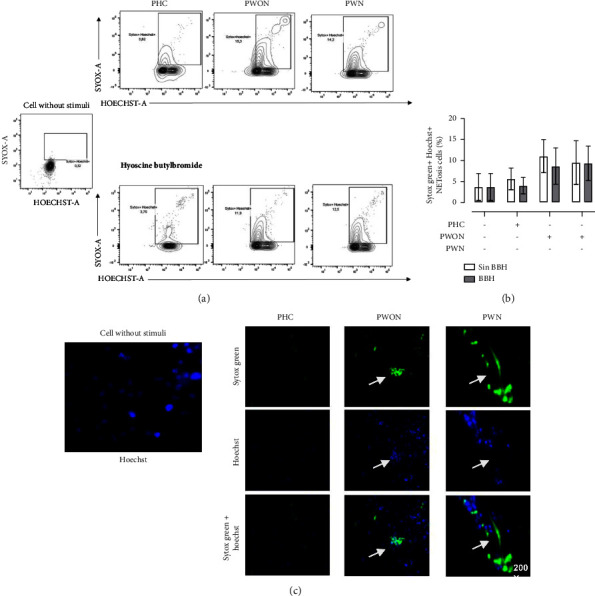
Role of muscarinic receptors in NETosis induced by sera from patients with LAC-associated vasculitis. Neutrophil-enriched suspensions from healthy controls were incubated with HBB (20 nM) for 10 min and then exposed to pooled sera (20%) from healthy controls (PHCs) and patients with LAC-associated vasculitis with (PWN) or without (PWON) GN for 6 h at 37°C. After incubation, cells were stained with Sytox Green and Hoechst 33342, washed, and analyzed by flow cytometry. (a) Representative dot plots and (b) column chart showing the percentage of double positive Sytox Green^+^ Hoechst 33342^+^ netting neutrophils generated in response to each treatment. Bars represent median values and error bars correspond to IQRs. ^*∗*^*p* ≤ 0.05; Kruskal–Wallis test. *n* = 4 independent experiments. (c) Representative images from confocal microscopy showing Sytox Green^+^ Hoechst 33342^+^ netting neutrophils. Magnification: 200x. *n* = 4 independent experiments.

To determine whether muscarinic receptors were also involved in NETosis induced by sera from patients with LAC-associated vasculitis, neutrophils were incubated with 20 *µ*L of 20 nM BBH for 10 min before exposure to PHC, PWN, and PWON pooled sera for 6 h at 37°C. After incubation, cells were stained with Sytox Green and Hoechst 33342, washed, and analyzed by flow cytometry. Preincubation with HBB did not change the percentages of netting neutrophils in cultures exposed to PWN (9.5% ± 1.12%–9.3% ± 0.89%) or PWON (11.02% ± 0.85%–8.6% ± 0.93%). No changes were observed in either of the cultures exposed to the PHC (5.6% ± 0.56%–3.8% ± 0.42%) (Figure 9).

## 4. Discussion

In the present work, neutrophils from healthy individuals exposed *in vitro* to cocaine, levamisole, and a cocaine-levamisole mixture released NETs containing MPO but not EN. Carmona et al. had reported levamisole-induced NETosis [13] and Lood and Hunges had demonstrated the release of NE-enriched NETs in response to cocaine or levamisole [17].

MPO release is a crucial event for NET formation. Indeed, Metzler et al. reported an increased risk of infection in MPO-immunodeficient individuals related to their inability to release NETs [18]. The presence of MPO but not EN observed in NETs released from neutrophils exposed to the cocaine-levamisole mixture suggests that this combination could induce NETosis through a different mechanism. Moreover, it would explain the predominance (92%) of circulating ANCAs and anti-MPO antibodies in the group of patients with LAC-associated vasculitis.

The cocaine-levamisole combination presented a synergistic effect on NETosis induction. This effect could be mediated by a levamisole-associated neuropharmacological mechanism that inhibits the enzyme monoamine oxidase and activates muscarinic receptors, thus increasing the transmission of dopamine signals and activation of NETosis-associated mechanisms [19, 20]. Another hypothesis attributes the synergistic effect of the cocaine-levamisole mixture to aminorex, a metabolite of levamisole that enhances the stimulant properties of cocaine. Prolonged consumption of aminorex has been associated with pulmonary hypertension and vasculitis [21, 22].

Inflammation depends on immunopathogenic and nonimmunopathogenic mechanisms. In the case of vasculitis, immunopathogenic mechanisms comprise those mediated by cells of the immune system, such as cytotoxic T cells directed against blood vessel components, the expression of adhesion molecules on endothelial cells, and the presence of antibodies that can form immune complexes that directly damage endothelial cells or are deposited on blood vessel walls [23]. In the present work, patients with LAC-associated vasculitis, confirmed by skin biopsy, were positive for ANCAs (92%), anti-MPO antibodies (64%), or anti-PR3 antibodies (28%). Because some patients (36%) had immune complex (IC)-mediated nephritis, it was hypothesized that ICs might cause the neutrophil cell death that characterizes LAC-associated vasculitis. Therefore, to determine whether ICs could induce NETosis, neutrophils were incubated with pooled sera from patients with nephritis (higher likelihood of circulating ICs) or patients without renal involvement (lower likelihood of circulating ICs). Pooled sera from patients with LAC-associated vasculitis induced only a slight increase in the percentage of netting neutrophils compared with cocaine and levamisole. Furthermore, no differences were observed between pooled sera from patients with or without GN. These results suggest that circulating ICs are not responsible for neutrophil cell death observed in patients with LAC-associated vasculitis and that other mechanisms are likely involved.

Muscarinic receptors are essential to neutrophil activation and could mediate NETosis induced by the cocaine-levamisole mixture. The results of neutrophil cultures preincubated with HBB, an M3 muscarinic receptor antagonist, prior to the addition of cocaine, levamisole, and the cocaine-levamisole mixture evidenced the involvement of these receptors in neutrophil cell death induced by each compound individually and when combined. Carmona et al. observed that levamisole-induced NETosis decreased in the presence of inhibitors of the muscarinic receptors M1 (Tele and UV) and M3 (4-DAMP). Other receptors that may participate in the induction of NETosis are Dectin-1, CD14, C1qR, Toll-like receptors (TLRs), and the Fc receptors that interact with ANCAs [24]. Carmona et al. also observed that PMA-induced neutrophil cell death was blocked in the presence of 4-DAMP and DARIF, an inhibitor of the RAF/MEK/ERK pathway [13]. In the present study, HBB did not inhibit the proNETotic effect of PMA but inhibited partially that of the cocaine-levamisole mixture, suggesting that the RAF/MEK/ERK pathway may be not involved in the induction of NETosis by cocaine and levamisole combined. These results support the administration of HBB as a therapeutic option for patients with LAC-associated vasculitis, as partial inhibition of NETosis could reduce clinical manifestations and limit the use of immunosuppressants and their associated adverse effects.

Several mechanisms of NETosis induction have been described, such as the activation of NADPH oxidase and the increased intracellular calcium levels. Activation of NADPH oxidase results in the synthesis of ROS that promotes the release of proteins from neutrophil cytoplasmic granules. The increase in intracellular calcium levels activates the peptidylarginin deiminase 4 (PAD4), an enzyme involved in histone citrullination. According to the results of the present work, cocaine, but not levamisole, induced ROS synthesis by neutrophils after 60 min of incubation. Moreover, the cocaine-levamisole mixture reduced ROS levels, suggesting an antagonistic effect of levamisole on cocaine-induced ROS generation. In contrast, Carmona et al. observed that levamisole induces the generation of ROS through the activation of the NADPH oxidase but not mitochondrial synthesis of the oxygen radicals, event that could be explained by the findings of [13].

As mentioned above, NETosis can be induced by an elevated intracellular calcium level that activates the PAD4 enzyme [25]. In the present study, BAPTA—an aminocarboxylic acid that chelates two calcium ions and thereby decreases free cytoplasmic calcium level—inhibited the NETosis induced by the cocaine-levamisole mixture. These preliminary results suggested that muscarinic receptors on the plasma membrane of neutrophils could mediate the induction of NETosis by the cocaine-levamisole mixture. Such receptors induce the activation of tyrosine kinases that phosphorylate phospholipase C. This enzyme hydrolyzes phosphatidylinositol bisphosphate to diacylglycerol and inositol triphosphate (IP3). The IP3 then binds to receptors in the endoplasmic reticulum to induce calcium release [26].

Elevated intracellular calcium levels in neutrophils activate the enzyme PAD4, which participates in chromatin decondensation, a hallmark of NETosis. Carmona et al. demonstrated that CI-amidine, a PAD4 inhibitor, decreased the percentage of netting neutrophil cells in cultures treated with levamisole [13]. In a murine model, Hemmers et al. observed that, in contrast to control mice, PAD4-deficient mice did not produce NETs after exposure to the influenza A virus. These results further supported that PAD4 is required to trigger neutrophil cell death by NETosis [27]. According to some studies, the citrullination of histones H3 and H4 is required for NET formation [28–30].

In addition, we evaluated whether proinflammatory cytokines could play a role in NETosis induced by the cocaine-levamisole mixture. Therefore, levels of IL-1*β*, IL-6, TNF-*α*, IL12-P70, and IL-8 were quantified in the supernatants of cultures exposed to each compound and the cocaine-levamisole mixture. Neutrophils only synthesized IL-8 in response to these treatments, in agreement with a study by Altstaedt et al. on the constitutive induction of IL-8 synthesis in neutrophils [31]. Likewise, neutrophils exposed to a pooled serum from patients with LAC-associated vasculitis showed a tendency to higher levels of IL-8 concentrations; these levels are the product of basal IL-8 concentrations and the IL-8 induced by serum.

Sera from patients with LAC-associated vasculitis can induce IL-8 synthesis, which may be due to their anti-MPO content. In Hsieh et al.'s study, neutrophils exposed to ANCA-MPO and ANCA-PR3 from ANCA vasculitis patients increased IL-8 levels in the presence of ANCA-MPO; these findings suggest that this antibody could be an inducer of IL-8 production [32].

In summary, cocaine and levamisole can induce NETosis through different pathways as follows: (i) activation of muscarinic receptors on the neutrophil cell membrane. This pathway is followed by the release of cytoplasmic granular proteins that translocate to the nucleus to decondense chromatin and generate NETs enriched in nuclear material and granular enzymes such as MPO. (ii) Increasing levels of intracellular calcium. Upon activation, IP3 generation would promote calcium release from the endoplasmic reticulum. In turn, intracellular calcium would activate PAD4 to mediate chromatin decondensation and subsequent release of NETs.

The muscarinic inhibitor HBB partially decreased NETosis induced by the cocaine-levamisole mixture, thus evidencing the involvement of muscarinic receptors in the induction of this form of cell death. Moreover, muscarinic receptors can also activate the intracellular calcium mobilization.

**Figure 10:**
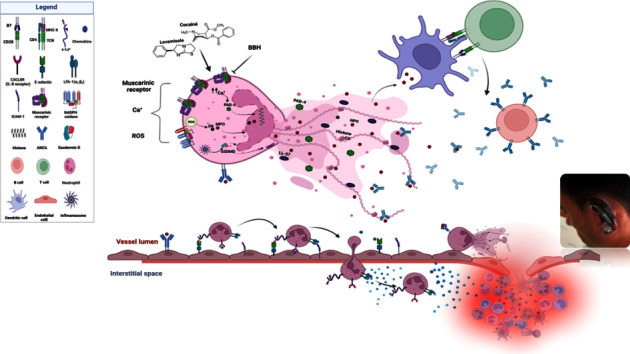
A model of the immunopathogenic mechanism underlying LAC-associated vasculitis.

Finally, we propose a model to relate NETosis induction by the cocaine-levamisole mixture to the clinical manifestations of LAC-associated vasculitis (Figure 10). According to this model, antigen-presenting cells would recognize the MPO on NETs and present it to CD4^+^T-cells. The activated T-cells would promote the activation of B-cells, leading to the synthesis of autoantibodies directed against MPO (ANCA-MPO). These autoantibodies, in turn, would amplify other cellular mechanisms such as the activation of more neutrophils through Fc receptors, the release of NETs, activation of endothelial cells to express adhesion molecules, decrease of blood flow, and binding of neutrophils to the endothelium. This way, an inflammatory and thrombotic environment would ensue and cause the characteristic clinical manifestations of LAC-associated vasculitis, such as necrosis of earlobes.

## 5. Conclusions

Cocaine and levamisole act synergistically to induce neutrophil cell death characterized by DNA and MPO extrusion. Although extracellular NE was not detected in neutrophils exposed to the cocaine-levamisole mixture, the detection of extracellular DNA and MPO, and the cellular characteristics evidenced by fluorescence microscopy, suggest that the combination of the two molecules induced neutrophil cell death by NETosis.

Sera from patients with LAC-associated vasculitis also induced DNA and MPO release from neutrophils with a pattern morphologically compatible with NETosis although to a lesser degree than the cocaine-levamisole mixture.

The mechanism underlying the NETosis induced by the cocaine-levamisole mixture seems to be independent of ROS generation. Levamisole did not induce ROS synthesis and even antagonized that induced by cocaine. Preliminary results suggest that NETosis induced by the cocaine-levamisole mixture depends on a calcium-dependent pathway.

HBB, a muscarinic receptor inhibitor, partially reduced the simultaneous effect of cocaine and levamisole on neutrophils, providing evidence for the involvement of these receptors in the induction of NETosis when both compounds are combined.

Finally, the present results propose flow cytometry as a valuable tool for the study of NETosis, given its simplicity, reproducibility, and low probability of data loss, as well as for being a more agile and higher throughput process.

## Figures and Tables

**Table 1:** Sociodemographic and clinical characteristics of patients with LAC-associated vasculitis (*n* = 25).

Sociodemographic data, *n* (%)			

Age (years)			34 [25–51]^*∗*^
Sex		Female	4 (16)
		Male	21 (84)
Substance abuse		Alcohol	16 (64)
		Marijuana	19 (71)
		Tobacco	19 (71)

Clinical and histopathological findings			

Necrosis of earlobes			20 (80)
Arthralgias/arthritis			16 (64)
Skin ulcers			9 (36)
Autoimmune hemolytic anemia			7 (28)
Nephritis		Immune complexes	9 (36)
		Other nephritis	6 (24)
Pseudovasculitis			4 (16)
Pyoderma gangrenosum			3 (12)
Vasculitis		Leukocytoclastic	2 (8)
Vasculopathies		Thrombotic	2 (8)

Laboratory findings			

Neutropenia			4 (16)
Hypocomplementemia		C3	13 (52)
		C4	3 (12)
Autoantibodies		ANAs	14 (56)
		ANCAs	24 (92)
		p-ANCA	9 (36)
		Anti-MPO	16 (64)
		Anti-PR3	7 (28)
Creatinine (mg/dl)		PWN	1.8 [1.1–4.0]^*∗∗*^
		PWON	0.7 [0.6–0.8]^*∗∗*^
Blood count	Hemoglobin	PWN	8.8 [7.6–12]^*∗∗*^
		PWON	10.6 [9.9–13.4]^*∗∗*^
	VSG	PWN	81 [52–103]^*∗∗*^
		PWON	87 [59.5–120]^*∗∗*^
	Platelets	PWN	342000 [239000–425000]^*∗∗*^
		PWON	407000 [236750–461500]^*∗∗*^
	Neutrophils	PWN	3310 [2100–5550]^*∗∗*^
		PWON	3190 [2697–6388]^*∗∗*^
	Lymphocytes	PWN	1180 [820–2300]^*∗∗*^
		PWON	1230 [675–2059]^*∗∗*^

^*∗*^Median and range. ^*∗∗*^Median and interquartile range.

## Data Availability

The data used to support the findings of this study are available from Dr. Carlos Muñoz-Vahos (carlmuvahos@gmail.com) upon request.
